# Dynamic brain states during encoding and their post-encoding reinstatement predicts episodic memory in children

**DOI:** 10.1101/2025.11.25.690570

**Published:** 2025-11-26

**Authors:** Yimeng Zeng, Sandhya P. Chakravartti, Srikanth Ryali, Shaozheng Qin, Vinod Menon

## Abstract

Episodic memory, the ability to rapidly learn and explicitly remember past events and experiences, plays a critical role in children’s academic learning and knowledge acquisition. The formation of lasting memories relies on the brain’s ability to dynamically organize its activity. How these neural configurations unfold moment-by-moment across encoding and offline phases remains poorly understood. To probe these dynamics, we applied a novel Bayesian Switching Dynamic Systems approach, a hidden Markov model with automatic state detection, to fMRI data from children performing scene encoding followed by an offline post-encoding rest. We identified four distinct brain states during encoding with unique activation modes between visual, medial temporal lobe, and frontoparietal and default mode network nodes. An “active-encoding” state with integrated visual-hippocampal and frontoparietal activity dominated encoding and predicted individual memory performance, while an inactive state negatively predicted performance. State transition dynamics revealed that flexible shifts into the active-encoding state enhanced memory formation, whereas transitions toward inactive states impaired it, demonstrating that memory success depends on dynamic neural flexibility. Critically, encoding states spontaneously reemerged during post-encoding rest. A “default-mode” state characterized by enhanced default mode network activity showed sustained maintenance during rest and robustly predicted memory outcomes, an effect specific to post-encoding, not pre-encoding rest. These findings establish that episodic memory emerges from coordinated brain state sequences bridging online encoding with offline consolidation, providing a computational framework for how moment-to-moment neural dynamics support memory formation in children. This work has broad implications for optimizing educational interventions and understanding developmental disorders affecting learning and memory.

## Introduction

Understanding how children's brains form lasting memories is fundamental to optimizing education and identifying learning disabilities [1-3]. Episodic memory, the ability to rapidly learn and explicitly remember past events and experiences [4], plays a critical role in children's academic learning and knowledge acquisition [2,5]. Episodic memory formation requires the brain coordinate activity across multiple distributed systems through flexible, moment-to-moment functional interactions during both online encoding and subsequent offline consolidation phases [6-8]. Despite its developmental importance, the dynamic neural mechanisms underlying this essential cognitive capacity remain unclear. Here, using a novel computational approach that identifies transient coordination patterns, or "brain states", we examine how the developing brain dynamically shifts between these states during learning and how these states are spontaneously reinstated during rest to support memory consolidation. Understanding these dynamic processes is critical for developing educational interventions that optimize learning and for identifying neural markers of memory difficulties in children.

Decades of research have identified key brain regions essential for episodic memory formation, revealing a hierarchical processing architecture [9,10]. Visual processing begins in ventral stream pathways from the occipital lobe, which extract visual features and object representations from incoming stimuli. This perceptual information is transmitted to the medial temporal lobe (MTL), particularly the hippocampus and parahippocampal gyrus, which serves as the critical binding hub for integrating diverse perceptual, spatial, and temporal elements into coherent, detailed episodic representations [11-14]. The lateral frontoparietal network provides top-down cognitive control and attentional modulation that selectively guides which information enters memory and how encoding processes unfold across time [15-17]. Additionally, the default mode network (DMN), particularly the medial prefrontal cortex (mPFC) and posterior cingulate cortex (PCC), plays a critical and dynamic role in episodic memory formation. These DMN regions are thought to undergo flexible reconfiguration, transiently integrating with taskpositive networks during encoding to support self-referential processing and contextual integration[18,19].

While prior research has provided essential insights into how episodic memory relies on individual brain regions, successful episodic memory formation critically depends on the temporal coordination between these systems. Brain regions must coordinate their activity through dynamic functional interactions, rapidly reconfiguring their activity and connectivity patterns in response to changing cognitive demands and stimulus characteristics. This dynamic network reconfiguration enables flexible adaptation to diverse task contexts – a capacity that is fundamental for efficient learning [20-22]. Investigations of memory systems in children have traditionally focused on role of individual brain regions [1-3], but how these systems interact dynamically during encoding and how they coordinate during offline consolidation to support memory formation remains largely unknown.

Fundamental questions remain unanswered: how do distributed brain regions involved in dynamically assemble into specific functional states during encoding? How do transitions between these states determine memory success? Understanding these dynamic assembly processes is critical because episodic memory formation likely depends on the brain's ability to flexibly coordinate distributed circuits into optimal network configurations to support memory formation, rather than sustained activation of individual regions. Conventional functional connectivity analyses or regional activation studies cannot detect the dynamic coordination underlying episodic memory, creating critical knowledge gaps. Novel computational approaches are needed to identify brain states that reflect the dynamic assembly of distributed neural circuits and to determine how these states contribute to encoding success and subsequent memory outcomes.

A second critical gap concerns the relationship between encoding dynamics and offline consolidation processes. Recent research has demonstrated that during awake rest or sleep, specific neural circuits automatically retrieve and reinstate memory traces – a process known as replay or reinstatement that is crucial for memory consolidation [6,7,23]. This reinstatement likely requires extensive dynamic coordination between brain areas to facilitate reorganization of memory representations into long-term storage [7,24,25]. However, a fundamental question remains: Do the specific brain states that emerge during encoding spontaneously re-occur during post-encoding rest, and does this reinstatement predict memory outcomes? Understanding how dynamic interactions among memory-related brain states evolve from encoding to post-encoding rest is critical for revealing the neural mechanisms underlying memory formation. This represents a key missing piece in our understanding of how episodic memories are formed, maintained, and consolidated across online and offline phases.

To address these critical gaps, we implemented a novel Bayesian Switching Dynamic Systems (BSDS) approach combined with graph-theoretic network analyses. BSDS implements a hidden Markov model with automatic relevance detection that determines the optimal number of brain states through unsupervised learning, overcoming the arbitrary parameter selection that constrains conventional approaches [26]. The BSDS model offers several key advantages: it identifies latent brain states and their temporal evolution without arbitrary parameter constraints, quantifies state lifetime and occurrence rates, and assesses dynamic transition patterns using moment-to-moment detection algorithms. Crucially, BSDS can probe mental processes that are internally organized rather than strictly constrained by external stimuli, making it ideally suited for capturing flexible, adaptive neural dynamics underlying memory formation [26,27].

Twenty-four typically developing children (ages 8-13) underwent an fMRI scan while performing episodic memory encoding of natural scenes, followed by a 6-minute post-encoding rest session and a subsequent recognition memory test with confidence ratings outside the scanner (**Figure 1**). We focused on 16 core regions spanning visual cortex, medial temporal lobe, frontoparietal networks, and DMN nodes that showed significant subsequent memory effects (remembered > forgotten contrast). We tested three primary hypotheses. First, BSDS would reveal dissociable brain states with distinct functional connectivity patterns among memory-related circuits during encoding, with occurrence of specific encoding-optimal states predicting individual memory performance. Second, dynamic transitions between brain states would be critical for memory formation, with transitions toward optimal encoding states enhancing performance and transitions toward suboptimal states impairing it. Third, and most importantly, brain states identified during encoding would spontaneously re-occur during post-encoding rest through reinstatement mechanisms, with offline state reactivation predicting memory consolidation success. This would provide direct evidence for systems consolidation through dynamic state reinstatement.

Our comprehensive framework provides the first systematic characterization and computational modeling of how dynamic brain states bridge online encoding with offline consolidation to support episodic memory formation in children. By revealing the specific brain state configurations and transitions that optimize learning, including the critical role of DMN-mediated consolidation states during rest, this work has broad implications for understanding memory function in children, for developing educational interventions that enhance memory formation, and enabling early identification of children at risk for learning disabilities.

## Results

### Episodic memory performance

We first validated that our experimental paradigm successfully engaged episodic memory processes in children, as evidenced by robust behavioral performance and confidence-based retrieval patterns. During fMRI scanning, participants encoded 100 images of buildings and scenes, followed by a post-scan memory retrieval test with confidence ratings (**Fig. 1A-1B**). We analyzed hit rates, confidence ratings, and their relationship to assess episodic memory formation. Children demonstrated successful episodic memory encoding with a significant hit rate ($t_{(23)} = 3.97$, $p < 0.001$) and they rated remembered items with higher confidence rating than forgotten ones ($t_{(23)} = 7.50$, $p < 0.001$) (**Fig. S1**). One-way analysis of variance (ANOVA) revealed a significant main effect of confidence on memory retrieval ($F_{(1,47)} = 15.03$, $p < 0.001$), with the strongest effect for highest confidence trials. These behavioral results confirm that our paradigm successfully engaged episodic memory processes in children, with confidence ratings serving as a reliable indicator of memory strength, providing a foundation for investigating neural mechanisms underlying successful encoding.

### Dynamic brain states during the episodic memory encoding phase

Episodic memory formation requires coordinated activity across distributed brain regions, yet conventional approaches cannot capture the dynamic functional interactions that support memory encoding. We applied Bayesian Switching Dynamic Systems (BSDS), a validated state-space modeling approach [26-28], to time series from 16 memory-related regions of interest, identified through subsequent memory effects (remembered > forgotten contrast; **Fig. 1C-D**). These regions spanned four characteristic functional modules including: visual-related ventral temporo-occipital cortex, medial temporal lobe system, and frontal-parietal networks area as well as default mode networks [29,30] (**Table.S1**). BSDS identified latent brain states with distinct amplitude patterns and functional connectivity signatures during episodic memory encoding.

BSDS first inferred four latent brain states with unique characteristics (**Fig. 2A).** State 3 ("active-encoding state") exhibited strong co-activation across visual areas, MTL system, and parietal regions while State 2 ("default mode state") showed peak activation in medial prefrontal cortex (MPFC) and posterior cingulate cortex (PCC). State 4 ("inactive state") displayed uniformly low amplitude across all regions, while State 1 showed intermediate activation patterns.

Subsequent functional connectivity analysis revealed that the active-encoding state (S3) exhibited significantly higher average connectivity during encoding (0.28) than all other states (paired *t*-tests: all ps < 0.001, FDR corrected; **Fig. 2B**). Graph theory analyses further demonstrated that the active-encoding state (S3) had significantly higher clustering coefficient and global efficiency compared to other states (all permuted ps < 0.05, FDR corrected; **Fig. 2C-D**).

These findings reveal distinct brain state configurations during encoding, with the active-encoding state characterized by optimal network integration and segregation properties. The high connectivity and efficiency of this state suggest it may represent a specialized neural configuration for successful memory formation, involving coordinated activity across visual processing, hippocampal binding, and attentional control systems.

**Brain state dynamics during the encoding phase predict memory performance**

To test whether these brain states reflect functionally meaningful neural configurations, we examined how their temporal dynamics – occupancy rates (OR), mean lifetime and state-to-state transition patterns – predict individual memory performance (Methods). Brain states showed markedly different occupancy patterns during encoding (**Fig. 3A**). The active-encoding state (S3) dominated with 38.39 ± 7.58% occupancy rate, significantly higher than all other states ($t_{(23)} > 5.43$, ps < 0.001, FDR corrected). Meanwhile, the mean lifetimes of the four states did not differ (all $ts_{(23)} < 1.47$, Ps > 0.86, FDR corrected). Critically, active-encoding state occupancy rate correlated positively with memory accuracy ($r = 0.47$, $p < 0.05$, FDR corrected), while inactive state occupancy showed a strong negative correlation ($r = -0.68$, $p < 0.01$, FDR corrected; **Fig. 3C**).

We then investigated whether state transitions during encoding contribute to episodic memory performance. State transition analysis revealed complex dynamics with prominent transitions toward active-encoding states (**Fig. 3D**). Notably, transitions to the active-encoding state were beneficial: both default mode state (S2) → active-encoding ($r = 0.49$, $p < 0.05$, FDR corrected) and inactive state (S4) → active-encoding transitions ($r = 0.48$, $p < 0.05$, FDR corrected) correlated positively with memory performance (**Fig. 3E**). Conversely, transitions toward the inactive state (S4) were detrimental, with incoming transitions showing negative correlations with memory accuracy (default mode state (S2) → inactive (S4): $r = -0.70$, $p < 0.01$; active encoding → inactive: $r = -0.63$, $p < 0.01$, FDR corrected; **Fig. 3F**).

These results demonstrate that successful episodic memory encoding depends not only on the presence of optimal brain states but also on the dynamic transitions between states to a more optimal brain state. The active-encoding state serves as a "hub" configuration that facilitates memory formation, while pathways leading to this state enhance performance and transitions away from it impair encoding.

**Recurrence of latent encoding brain states during the post-encoding rest phase supports memory consolidation**

Systems consolidation models propose that memory traces encoded during learning are reactivated during subsequent rest periods to facilitate long-term storage [31-33]. Next, we examined whether brain states identified during encoding would re-emerge during post-encoding rest, and whether this reinstatement would be associated with memory performance. We applied the BSDS model trained on encoding data to decode brain states during the pre- and post-encoding rest session using maximum log-likelihood estimation (**Fig. 4A, Methods**).

We first investigated the association of state distribution between encoding and post-encoding rest. By calculating correlations between individual-paired state distributions (Methods), we found a significant correlation between the encoding and post-encoding rest phases, when compared with shuffled paired state distributions (r = -0.62, permuted p<0.05), suggesting that such correlation is individual specific (**Fig. S3A**). Notably, such individual-specific associations validate our decoding result. Further entropy analyses based on states distribution (Methods) revealed significantly higher entropy during post encoding rest compared with the encoding session (paired t-test, $t_{(23)} = 6.32$, $p < 0.001$), suggesting a substantial state distribution shift, from a task-prioritized distribution toward a more evenly distributed mode during post-encoding rest (**Fig. S3B)**.

Next, we compared the state dynamicity shift for each brain state and as indicated in **Fig 4B**, we found that the active-encoding state (S3) which yielded the highest occupancy rate during encoding phase decreased substantially during the post-encoding rest phase (paired t-test: $t_{(23)} = -6.02$, $P < 0.001$, FDR corrected), and the intermediate state (S1) became the dominant state during post-encoding rest compared with other states (all $ts_{(23)} > 6.19$, $Ps < 0.001$, FDR corrected). In addition, the mean lifetime of all states decreased substantially during post-encoding rest (**Fig. 4C**). Further comparison of state occupancy rate and mean lifetime between pre- and post-rest revealed a significantly higher occupancy rate ($t_{(23)} = 2.12$, $p = 0.045$, uncorrected) and a marginally higher mean lifetime ($t_{(23)} = 1.78$, $p = 0.089$, uncorrected) of active-encoding state (S3) during post-encoding rest compared with pre-encoding rest (**Fig. S4**), suggesting an encoding-induced state dynamic reorganization during post-encoding rest. In terms of transition dynamics, we observed a substantial shift from encoding toward post-encoding rest (**Fig. 4E**), as indicated by a significant Frobenius norm difference between the two transition networks (permuted-$p < 0.001$). But we didn't observe such shift in transition dynamics between pre- and post- rest (permuted-$p > 0.28$). Together, these results indicate a systematic shift of brain state dynamics from encoding toward a post-encoding reinstatement process.

Finally, we investigated whether the reinstatement of specific encoding states during rest predicted subsequent memory performance. As recent studies have shown that the activity of the default mode network coincide with potential neural replay to further support learning or memory consolidation [19,34], we first examined the default mode state (S2), characterized by both high activation in MPFC and PCC nodes, which showed the strongest reinstatement effects. We found that its mean lifetime during rest correlated robustly with memory accuracy ($r = 0.45$, $p = 0.026$; **Fig. 4D**), a relationship that was absent in other brain states (all $ps > 0.42$). Notably, we found such reinstatement effect only occurred during post-encoding rest as occupancy rate in pre-encoding rest did not exhibit similar reinstatement effect ($r=0.24$, $p = 0.25$), indicating that such reinstatement is specific to post-encoding rest. Furthermore, we investigate whether transitions of the default mode state are also predictive of memory performance. As expected, the stability of this state, but not other states, during post-encoding rest—measured by self-transition probability—showed the strongest correlation with memory performance ($r = 0.54$, $p < 0.001$, FDR corrected; **Fig. 4F**). Again, we did not find such memory-predictive reinstatement occurred during pre-encoding rest either, again validating that such reinstatement could only occurred for post-encoding rest rather than pre-encoding phase.

Together, these findings demonstrate that successful episodic memory formation extends beyond initial encoding to include dynamic offline processes that strengthen and reorganize memory representations through systematic neural replay.

## Discussion

We examined how children's brains dynamically organize into distinct coordination patterns or "brain states", during memory formation and how these states are reinstated during rest to support consolidation. Using a novel Bayesian state-space modeling approach, we identified four distinct brain states with unique activation and connectivity signatures during episodic memory encoding of scenes. An "active-encoding state" characterized by integrated visual-hippocampal-parietal activity dominated successful encoding. Critically, flexible transitions into the active-encoding state enhanced memory formation. Most importantly, we discovered that encoding states spontaneously reinstated during post-encoding rest, with a "default-mode state" involving mPFC and PCC showing sustained reactivation that robustly predicted memory outcomes. These findings reveal that successful memory formation in children depends on dynamic neural flexibility, characterized by the ability to shift into optimal states during learning and to spontaneously reactivate consolidation-related states during rest. Results advance our understanding of how moment-to-moment brain dynamics support memory across online and offline phases.

### Dynamic brain states reveal moment-to-moment neural flexibility during the memory encoding phase

We addressed a fundamental challenge understanding how distributed brain regions coordinate their activity over time to support episodic memory formation in children. While extensive evidence demonstrates that memory depends on coordinated interactions between MTL and distributed cortical networks [9,12,35-38] – conventional static connectivity approaches cannot capture the dynamic network reconfigurations underlying successful encoding. Leveraging dynamic state-space hidden Markov models [26], we identified four distinct brain states with unique activation and connectivity signatures that emerge during memory encoding.

Our core findings that a specific "active-encoding state" characterized by enhanced activity in visual, hippocampal and frontoparietal networks dominates successful encoding and strongly predicted individual memory performance. provides direct evidence for optimal network configurations that facilitate memory formation. This configuration aligns with extensive evidence showing that visual cortical and hippocampal activity during scene encoding predicts better memory recall [39,40], and that lateral frontoparietal coordination guides selective attention to information that the hippocampus binds into relational memory traces [11,12,41,42] [43,44]. Our findings thus provide dynamic network-level evidence for established component-process and attention-to-memory models [9,17,45]. Critically, the “active-encoding state” state exhibited high global efficiency and clustering coefficient, indicating an optimal balance between network integration and specialized processing. This architecture facilitates efficient binding of visual features into coherent episodic representations, consistent with models proposing that fluctuations between integration and segregation, particularly in frontoparietal-hippocampal systems, support transitions between perceptual processing and relational binding [26,35,46].

Notably, we identified two additional brain states with distinct functional profiles. An inactive state characterized by uniformly low amplitude across all regions showed strong negative correlations with memory performance, revealing that successful encoding depends not only on achieving optimal configurations but also on avoiding maladaptive states that impair memory formation. Notably, we detected a default-mode state involving selective activation of mPFC and PCC, core nodes of the DMN known for their role in internal mentation, self-referential processing, and memory integration[18,19]. The emergence of this state during encoding is consistent with recent theoretical frameworks proposing that DMN regions undergo flexible reconfiguration, transiently integrating with task-positive networks to support contextual binding and encoding processes[19,47]. This challenges traditional views of the DMN as solely a "task-negative" network and instead suggests dynamic, state-dependent involvement in memory formation.

Moreover, the presence of this default-mode state during encoding, coupled with the absence of strict trial-by-trial alignment between states and task events, indicates that encoding dynamics are partly internally organized rather than purely stimulus-driven. This internal organization likely reflects fluctuations in attention, engagement, self-referential processing, and encoding strategies that critically determine memory outcomes [26,48] [18,49,50]. Together, these findings demonstrate that dynamic brain states reflect the full spectrum of cognitive processes determining memory outcomes from optimal active-encoding and DMN-mediated configurations to detrimental inactive patterns.

Finally, our analysis of state-to-state transitions revealed another fundamental mechanism: successful encoding depends not only on achieving optimal brain states but also on the flexibility to transition into these states when needed. Transitions toward the active-encoding state from both default-mode and inactive states positively correlated with memory performance, while transitions toward the inactive state were detrimental (**Fig. 3E**). These transition patterns reflect rapid neural reorganization through dynamic assembly of multiple circuits to support learning and flexible information access [51-53], consistent with evidence that stable yet flexible hippocampal states maintained by attentional and prefrontal modulation predict successful encoding [54-56]. This dynamic flexibility extends findings from motor skill learning and working memory showing rapid transitions between network integration and segregation [57-59], suggesting that memory formation emerges from the brain's capacity for rapid, adaptive network reorganization rather than sustained activation of fixed circuits.

**Encoding state reinstatement during the post-encoding rest phase reveals offline consolidation mechanisms**

Systems consolidation models propose that memory traces are spontaneously reactivated during rest to facilitate long-term retention [10]. Our state space modeling revealed two key findings during the post-encoding rest. First, all four encoding states spontaneously emerged during rest, with the active-encoding state showing significant increases during this offline period. This demonstrates that encoding-related network configurations are not simply taskdriven responses but represent stable coordination patterns that persist and reactivate spontaneously. Second, the "default-mode" state, characterized by enhanced activity in mPFC and PCC, demonstrated the strongest reinstatement effects, with its stability during post-rest robustly predicting memory performance. This provides direct evidence for systems consolidation through state-specific reinstatement, advancing beyond previous studies focused on regional reactivation patterns [60,61]. The dissociation between encoding-dominant and rest-dominant states reveals complementary mechanisms: the active-encoding state supports initial memory formation via sensory-hippocampal integration, while the default-mode state orchestrates offline consolidation through sustained mPFC and PCC activity. This temporal specialization demonstrates a critical principle of memory formation: different network configurations are optimized for different cognitive phases, requiring coordinated handoffs between states rather than simple persistence of encoding patterns.

This principle aligns is supported by converging evidence. Sustained hippocampal-neocortical coupling increases during post-encoding rest [62] and hippocampal replay events co-occur with strong DMN hub activation [34], suggesting that successful consolidation relies on coordinated interactions between memory encoding systems and DMN-mediated integration networks [63]. Our findings extend this framework by demonstrating state-level dynamics: the default-mode state that appears transiently during encoding, where it likely supports contextual integration, becomes substantially more prominent and sustained during offline rest, where it orchestrates memory stabilization and consolidation. This dual, temporally specialized role of DMN regions reconciles seemingly contradictory findings about DMN involvement in memory. Rather than being purely "task-negative," DMN regions undergo flexible, state-dependent reconfiguration (Menon, 2023): transiently integrating with task-positive encoding networks when contextual binding is needed, then dominating during offline periods to orchestrate sustained consolidation through mPFC-PCC coordination. Indeed, our observed reinstatement and sustained activation of the default-mode state during post-encoding rest provides direct empirical support for this framework, demonstrating that DMN-mediated network dynamics are fundamental for memory stabilization, integration, and adaptive updating during offline consolidation [19,64,65].

## Conclusions

Our study advances understanding of episodic memory by revealing how dynamic brain state sequences bridge online encoding with offline consolidation. We demonstrate that successful memory formation requires: (1) achieving optimal network configurations during encoding, (2) flexible transitions between states based on cognitive demands, and (3) systematic reinstatement of consolidation-related states during rest. These findings establish dynamic flexibility of brain states as a core principle of memory formation and provide a new framework for understanding how moment-to-moment brain state dynamics support learning and memory in the developing brain. This work opens new avenues for educational interventions and clinical applications.

## Methods

**Participants.** A total of 29 healthy children (16 girls and 13 boys) participated in this study. They were recruited from the San Francisco Bay Area. All participants were right-handed, had no history of neurological or psychiatric diseases, and were not currently taking any medications. To minimize age-related variability, children were selected from an age range of 8 to 13 (mean age: 11.03 ± 1.52). All participants had intelligence quotients (IQ) above 95 and below 145, as measured by the Wechsler Abbreviated Scales of Intelligence (WASI). Verbal, performance and full-scale IQ scores were normalized according to each participant's age. The study protocol was approved by the Stanford University Institutional Review Board. Written informed consent was obtained from each participant as well as the child's legal guardian prior to their participation. Participants with root mean squared head motion (i.e., RMS) exceeding one voxel's width during MR scanning were excluded from further analyses (5 participants), resulting in 24 child participants in the final study analysis.

**General experimental procedures.** At the start of the experiment each participant underwent a 6-minute pre-encoding resting state scan, and was instructed to close their eyes, let their minds wander. Following this scan each participant performed one episodic memory encoding task during fMRI scanning, followed by a post-encoding resting state scan wherein participants were instructed to imagine themselves in each scene and memorize the images for subsequent testing. Outside of the MRI scanner participants performed an episodic memory retrieval task (**Fig. 1A-B**). Details of the two memory tasks are described below. Participants underwent an event-related fMRI while performing the memory encoding task. The event-related fMRI experiment was designed to examine changes in trial-by-trial neural pattern stability with development in terms of the "overlapping waves" model [66]. The task consisted of 100 trials in total. Each trial consisted of an image presented at the center of screen for 3 seconds, followed by a fixation period jittering from 2.0 to 6.0 seconds. Participants were asked to press a button indicating whether the picture was of a building or a natural scene. Half of the trials consisted of images of buildings and half of images of natural scenes. The order of the 100 building and natural scene trials was pseudo-randomized across participants with the standard addition and control additional problems always interleaved by a low-level fixation period. Participants performed two experimental runs of the memory encoding task (we combined these two encoding tasks into one and assigned it as 'the encoding phase'). The length of each experimental run was 6 mins and 30 secs. The other settings were identical with the block design fMRI task.

**Retrieval task.** In the memory retrieval task, participants viewed 200 images of buildings and natural scenes, 100 of which they had studied during the memory encoding task and 100 of which they had not. Each trial consisted of an image presented at the center of screen for 3 seconds during which participants indicated via a "Yes" or "No" button press whether or not they had previously studied the picture during the encoding task. The following screen asked participants to indicate, again via a button press, how sure they were of their choice, using the following scale: "Very,", "Sure," "A little," "Unsure." A fixation period jittered from 2.0 to 6.0 seconds followed. As in the encoding task, half of the trials consisted of images of buildings and half of images of natural scenes. Participants performed four experimental runs of the memory retrieval task. The length of each experimental run was 6 mins and 30 secs. For each participant, we computed the rates of "hits," calculated as the proportion of studied images that were correctly identified as being seen during the encoding task; "misses," proportion of studied images that were incorrectly identified as being novel or unseen during the encoding task; "false alarms," proportion of unstudied images incorrectly identified as studied; and "correct rejections," proportion of unstudied images correctly identified as novel. From these rates, we also computed hit and miss as a function of confidence (**Supplement Fig. 1**) and participant's overall memory accuracy as the proportion of correct responses (hits and correct rejections) minus incorrect responses (misses and false alarms).

**fMRI data acquisition.** Whole brain functional images were acquired from a 3T GE Signa scanner (General Electric, Milwaukee, WI) using a custom-built head coil with a T2*-sensitive gradient echo spiral in-out pulse sequence based on blood oxygenation level-dependent (BOLD) contrast (12). Twenty-nine axial slices (4.0 mm thickness, 0.5 mm skip) parallel to the AC–PC line and covering the whole brain were imaged with the following parameters: volume repetition time (TR) 2.0 sec, echo time (TE) 25 ms, 80° flip angle, matrix size 64 × 64, field of view 200 x 200 mm, and an in-plane spatial resolution of 3.125 mm. To reduce blurring and signal loss arising from field inhomogeneity, an automated high-order shimming method based on spiral acquisitions was used before acquiring functional images. A linear shim correction was applied separately for each slice during reconstruction using a magnetic field map acquired automatically by the pulse sequence at the beginning of the scan.

**fMRI data preprocessing and time series extraction.** Images were preprocessed using Statistical Parametric Mapping (SPM8, http://wwwfil.ion.ucl.ac.uk/spm). The first eight volumes were discarded for stabilization of the MR signal. Remaining functional images were realigned to correct for rigid-body motion. Subsequently, images were slice-timing corrected, normalized into a standard stereotactic space, and resampled into 2 mm isotropic voxels. Finally, images were spatially smoothed by convolving an isotropic 3D-Gaussian kernel (6-mm full width at half maximum). Then, to extract time series for later BSDS model training, we first select candidate region of interests by contrasting neural activity for encoding trials later remembered with later forgotten (**Fig. 1C**). By doing so, we selected 16 ROIs covering MTL systems, visual-related areas and frontal-parietal regions as well as default mode hubs (**Supplementary Table. S1**). Each ROI was a 5-mm radius sphere centered at the corresponding peak voxel of each node. For encoding and post-encoding rest session, time series of the mean was extracted from each ROI and were high- pass filtered at 0.008 Hz after which the white matter and cerebrospinal fluid, as well as 6- head movement signals were further used as regressor to remove out from time series. Finally, the time series was linearly detrended and normalized before conducting model training.

**Bayesian Switching Dynamical Systems (BSDS) to infer brain dynamics during encoding.** We implemented an advanced Bayesian-based brain state detection model called BSDS to infer brain state during episodic memory task and decode latent state evolution during subsequent post-encoding rest. This model has been used in several recent studies and has the potential to detect cognitive state and pathology of both animal and human brain signals [26,27]. A detailed theoretical model is provided in our previous study [26]. BSDS could infer brain state or brain network dynamic configuration given a set of ROIs based on the time series. Its outputs consist of 1) Group-level brain state characterization of the state mean amplitude and covariance matrix across nodes. 2) Individual-level state dynamic metrics include occupancy rate and mean lifetime. 3) Group-level inter-state transitions among brain states via model-based estimation.

**Bayesian Switching Dynamical Systems (BSDS) to decode brain dynamics during pre- and post-encoding rest.** A unique feature of BSDS is that it could implement the trained model to decode untrained brain states via log-likelihood estimation. Based on this, we leverage the BSDS model trained on encoding session to investigate the brain state dynamics during pre- and post-encoding rest. After the model inference, we implemented the maximum-win principle together with threshold (0.1) for z-scored likelihood to determine the state evolution for pre- and post-encoding rest. Based on the decoded brain state sequence, we computed the occupancy rate and mean lifetime of each brain state during pre- and post-encoding rest. We also quantified transition dynamics between states during rest based on the heuristic transition estimation via observed transition behaviors for each participant during rest session.

**Transition network graph analysis.** For transition networks among brain states during encoding and post-encoding rest. We first obtained individual-level estimated transitions between brain states and depicted group-level graph network of transition by averaging each specific transition path on group-level. We excluded average transition values below 10% prior to visualization and the thickness of edges between brain states represents the relative strength of state transitions. To depict topographical patterns of each latent state, we conduct graph analysis based on Brain Connectivity Toolbox [67]. Negative covariance value in each covariance matrix is excluded before conducting network analyses. We computed global efficiency and clustering coefficient given group-level covariance matrix for each brain state. We conducted a permutation test to determine the statistical significance of differences in network properties between each pair of brain states. Consistent with previous study [68,69], we circularly shifted state assignment for each participant and computed the corresponding global efficiency and clustering coefficient respectively 5000 times. Then, we obtained null distribution of difference in global efficiency and clustering coefficient between each paired brain states. Finally, we determined the p-value by dividing the number of times the actual difference exceeded the null distribution of correlations.

**Statistical comparison of transition differences.**

To test whether the overall transition structure differed between encoding and post-encoding rest, we applied a within-subject sign-flip permutation test based on the Frobenius norm of the group-averaged difference matrices. For each participant, we calculated the difference between transition matrices. The test statistic was defined as the Frobenius norm of the group-averaged difference matrix, representing the overall magnitude of transition change. Then we generated null distribution via randomly flipped each participant's difference matrix corresponding to a random exchange of condition labels before calculating Frobenius norm for the group-averaged difference matrices. The $p$-value was determined as the proportion of permuted statistics greater than or equal to the observed value (permutation times: 5000).

**State-memory correlation analyses.** To test the association between brain state dynamics and corresponding individual memory performance, we conduct spearman correlation analyses for each brain state metric (occupancy rate/ mean lifetime/ state-to-state transitions) and memory accuracy both during the encoding phase and pre- and the post-encoding rest phase.

**Entropy analysis.** To quantify systematic changes in brain state dynamics from encoding to post-encoding rest, we computed the entropy value based on distribution of state occupancy rates as a representative metric. According to information theory[70,71], entropy measures the uncertainty of a stochastic system—here, a more evenly distribution of states corresponds to higher entropy, whereas a more skewed distribution indicates lower entropy. In the present study, we calculated the entropy based on the probability of each state's occupancy rate by the following formula:

$$H(x) = -\sum_{i \cong 0} p(x)\, log2\, p(x)$$

Where p(x) is the probability of each brain state's occupancy.

**Individual-paired state distribution correlation.** To investigate the subject-specific association in states distribution between encoding and post-encoding rest, we conducted a group-level correlation analysis. We first concatenated the state distributions (represented by their occupancy rate) across all subjects for both the encoding phase and their pairing in the post-encoding rest phase. We then computed the correlation between the two phases. To avoid collinearity (because OR's sum is equal to 1), we included only the first 3 brain states in the analysis. Statistical significance was assessed via a permutation test. To be specific, we randomly shuffle the pairing between encoding phase and post-encoding rest and compute the state distribution correlations 5000 times, thereby generating a null hypothesis distribution. P-value was determined by dividing the number of times the actual correlation is lower than the correlations in the null distribution.

**Acknowledgements.** This research was supported by a National Institutes of Health Career Development Award to SQ (K99MH105601), the National Institutes of Health to V.M. (MH084164), the National Natural Science Foundation of China (31522028, 81571056) and the Fundamental Research Funds for the Central Universities (2024-JYB-XJSJJ020).

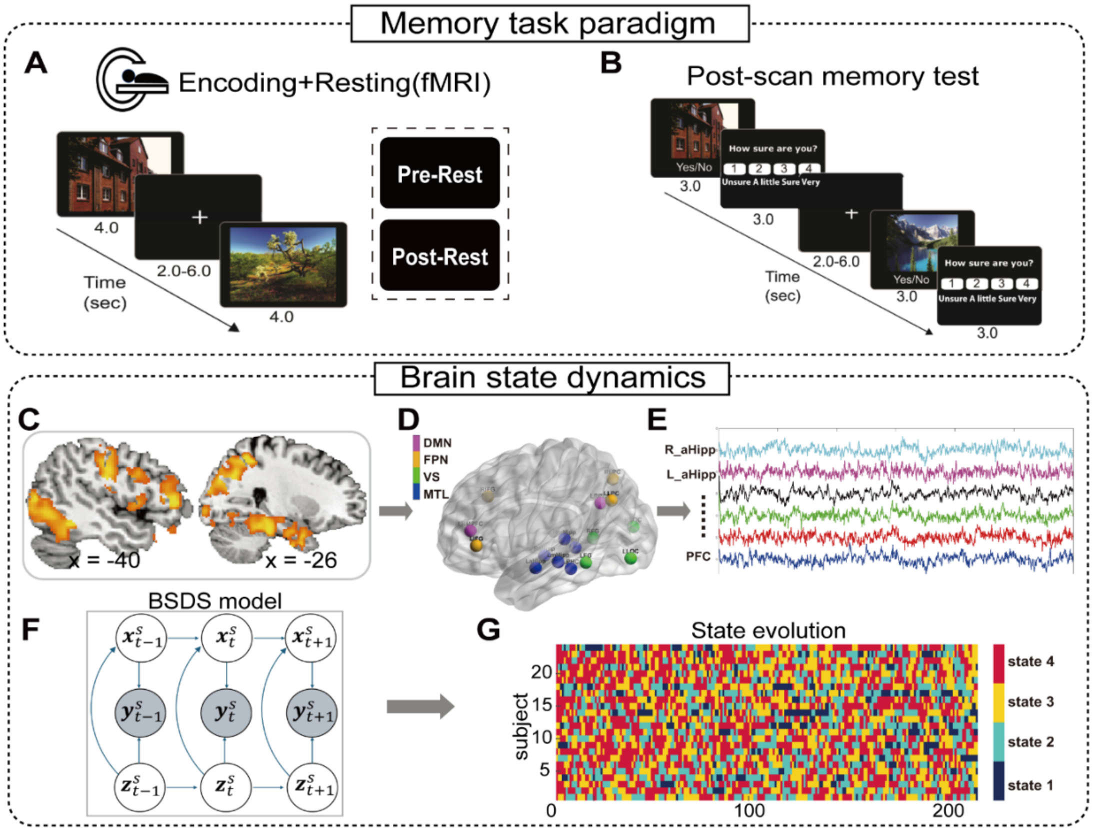

**Figure 1. Experimental design and dynamic brain state modeling framework (a) episodic memory task paradigm.** During fMRI scanning, participants encoded 100 images of buildings and natural scenes (3 seconds each, jittered fixation 2-6 seconds), followed by a 6-minute post-encoding rest period. Participants were instructed to imagine themselves in each scene and memorize the images for subsequent testing. **(B) Post-scan memory assessment.** Outside the scanner, participants completed a memory retrieval test with confidence ratings using a 4-point scale ("Unsure" to "Very Sure") to assess memory strength and subsequent memory effects. **(C) Memory-related brain regions.** Lateral view showing significant activation clusters (remembered > forgotten contrast) across ventral visual areas, medial temporal lobe, and frontal-parietal regions that served as the foundation for dynamic state modeling. **(D) Regions of Interest for state space modeling.** Sixteen core regions exhibiting significant subsequent memory effects, spanning visual association (VS) (fusiform, lateral occipital complex), memory encoding-related medial temporal lobe (MTL) (hippocampus, parahippocampal cortex), and cognitive control (frontal-parietal network) systems and default mode networks (DMN) (medial prefrontal and posterior cingulate cortex). ROIs are separately color coded by system. **(E) Time series extraction.** Representative raw BOLD time series from memory-related ROIs (Right anterior hippocampus, left anterior hippocampus, posterior cingulate cortex and medial prefrontal cortex) during encoding, preprocessed and normalized for dynamic brain state modeling. **(F) Bayesian Switching Dynamic Systems model.** Schematic illustration of the unsupervised BSDS framework that identifies latent brain states from multi-region time series without arbitrary parameter constraints. **(G) State evolution visualization.** Color-coded temporal evolution of four distinct brain states inferred by BSDS across all participants during memory encoding, revealing moment-to-moment state transitions at TR-level resolution.

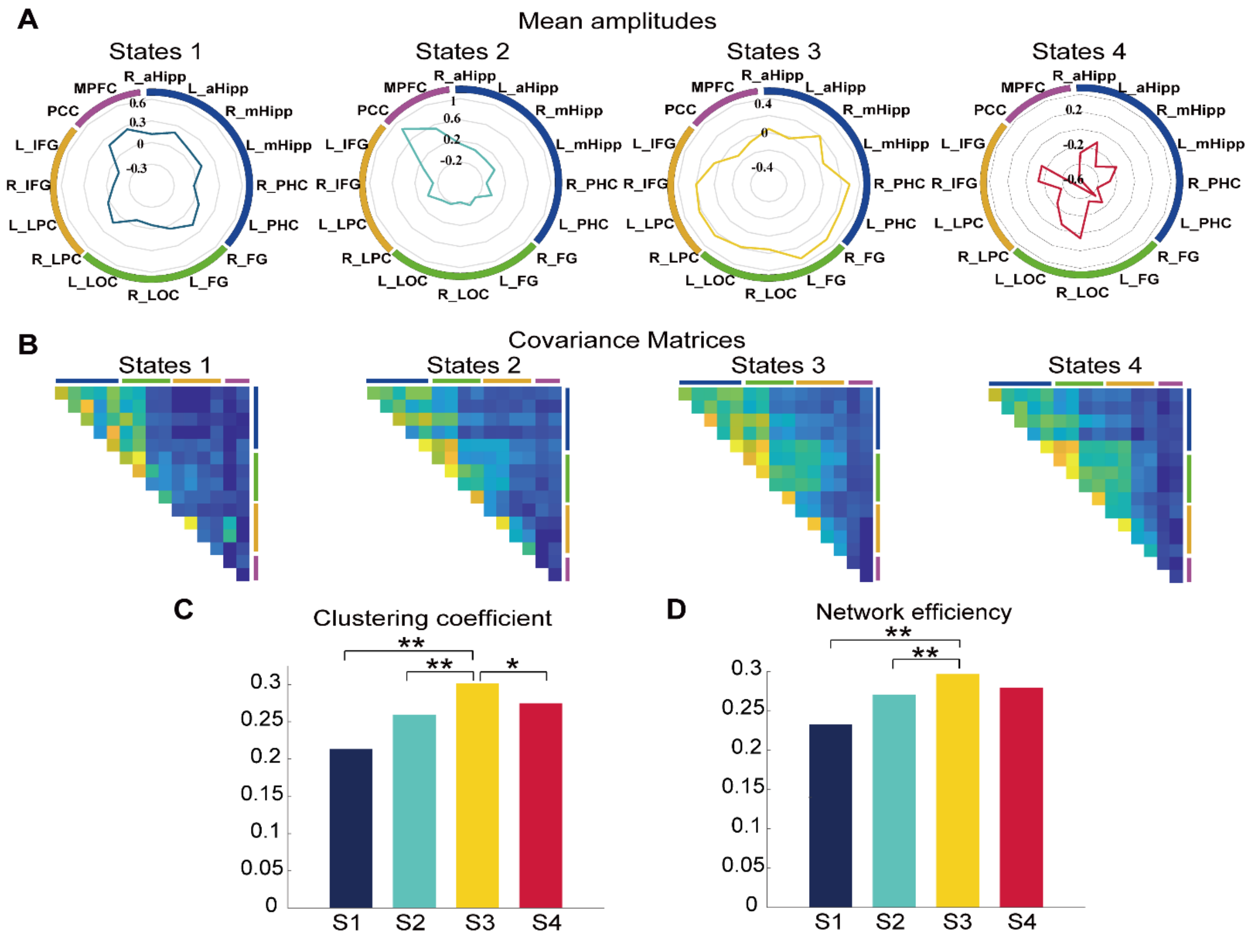

**Figure 2. Characterization of dynamic brain states during memory encoding. (a) State-specific activation patterns.** Polar plots depicting mean amplitude profiles across 16 memory-related regions for each of the four brain states identified by BSDS. State 3 (core-encoding state) shows prominent activation across visual areas, MTL system, and frontal-parietal regions. State 2 (default mode state) exhibits selective activation in medial prefrontal and posterior cingulate cortex. State 4 (inactive state) displays uniformly low activation, while State 1 shows intermediate patterns. **(B) Functional connectivity matrices.** Group-level covariance matrices revealing distinct connectivity signatures for each brain state. The active-encoding state (S3) demonstrates the highest overall functional connectivity, significantly exceeding all other states. **(C-D) Network topology analysis.** Bar graphs show clustering coefficient and global efficiency metrics derived from connectivity matrices. The active-encoding state exhibits significantly higher clustering coefficient and global efficiency compared to other states, indicating optimal balance between network integration and segregation for memory formation. Error bars represent standard error. Notes: *$p < 0.05$, **$p < 0.01$.

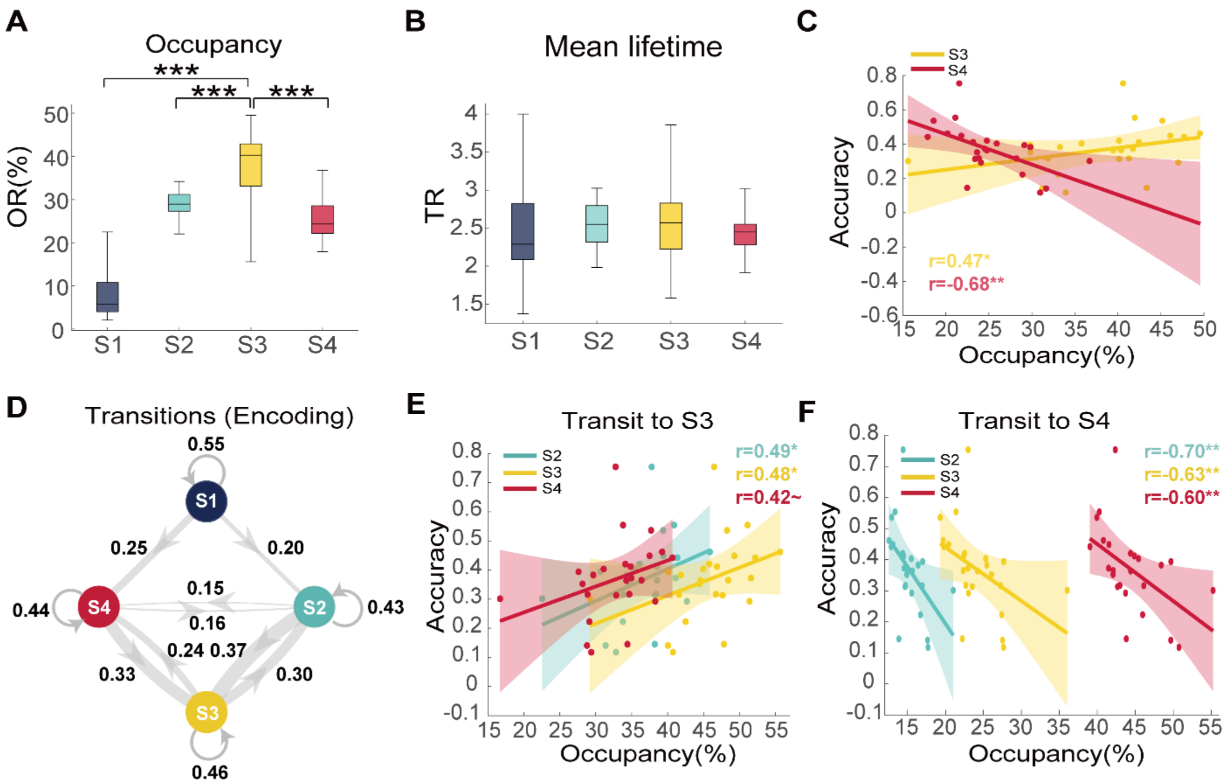

**Figure 3. Brain state dynamics predict individual memory performance. (A-B) State temporal properties.** Box plots show occupancy rates and mean lifetimes for each brain state during encoding. The active-encoding state dominates with 38.39% occupancy, significantly higher than all other states. **(C) Memory performance correlations.** Scatter plots revealing that core-encoding state occupancy correlate positively with memory accuracy, while inactive state occupancy shows strong negative correlation. **(D) State transitions.** Network diagram illustrates transition probabilities between brain states during encoding, with edge thickness representing transition strength. Prominent bidirectional transitions occur between core-encoding, high-order control, and inactive states. **(E-F) Transition-performance relationships.** Scatter plots demonstrating that transitions toward the active-encoding state (S2→S3; S4→S3) enhance memory performance, while transitions toward the inactive state (S2→S4; S3→S4) impair performance. Self-transitions of the inactive state also negatively predict memory. Dashed lines indicate 95% confidence intervals; solid lines show best linear fit. Notes: ~$p < 0.10$,*$p < 0.05$, **$p < 0.01$, ***$p < 0.001$.

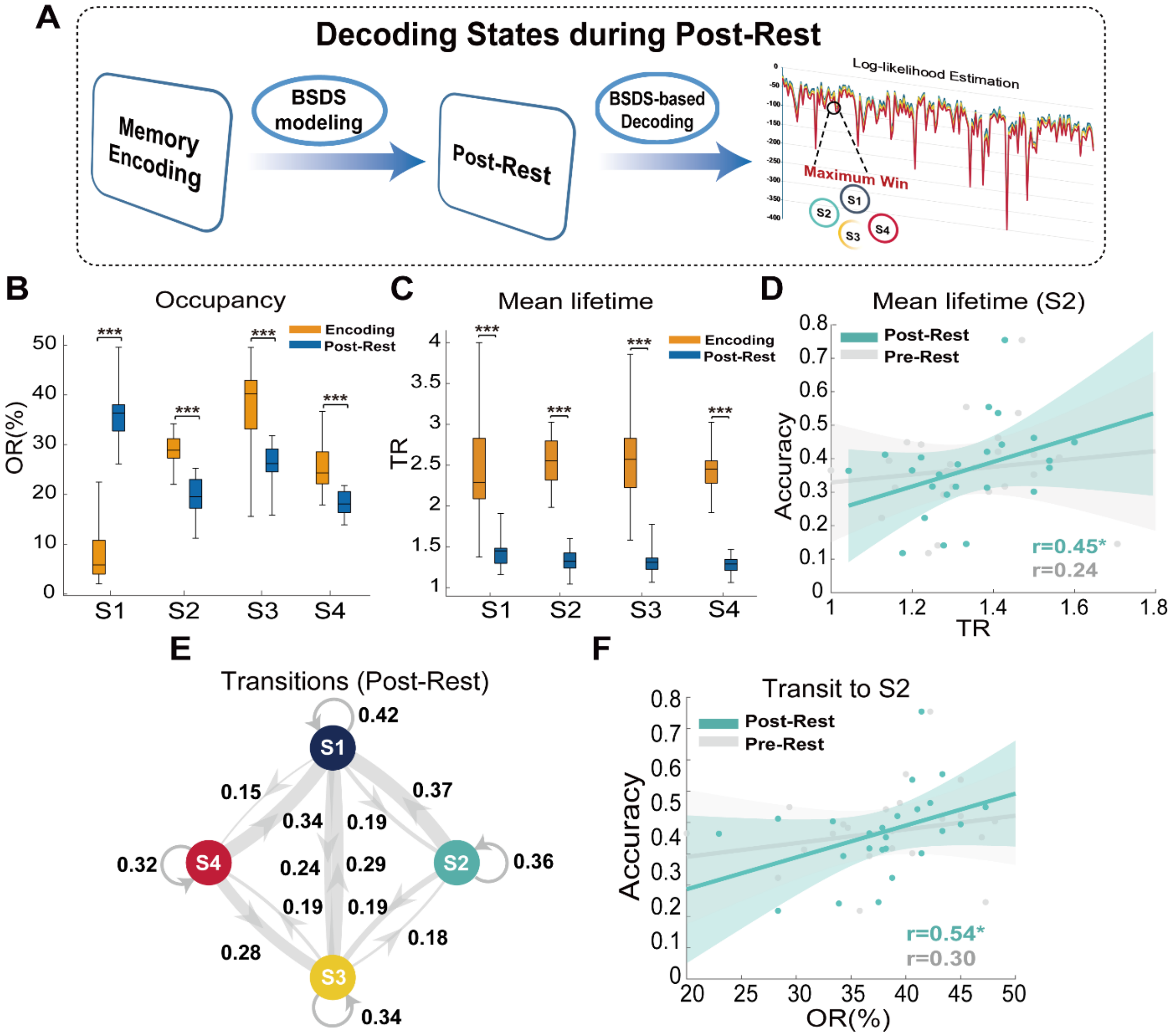

**Figure 4. Encoding state reinstatement during post-encoding rest supports memory consolidation. (A) State decoding framework.** Schematic workflow for detecting encoding state re-occurrence during post-encoding rest using the BSDS model trained on encoding data. Maximum log-likelihood estimation determines state assignment at each time point during rest. **(B) State occupancy shifts.** Bar graph comparing state occupancy between encoding and rest phases. All task-related states (S2, S3, S4) decrease significantly during rest, while State 1 increases substantially, reflecting the state dynamic shift during rest **(C)Mean lifetime shifts.** Bar graph comparing mean lifetime between encoding and post -encoding rest phases. All states decrease significantly during rest. **(D) Memory-predictive reinstatement.** Scatter plot showing that the mean lifetime of the high-order control state (S2) during post-encoding rest, but not pre-encoding rest, correlates strongly with memory accuracy, indicating that sustained activation of this MPFC-PCC network supports offline consolidation. **(E) Rest transition dynamics.** Network diagram showing altered transition patterns during rest, with decreased self-transitions and increased inter-state transitions compared to encoding. **(F) Consolidation state stability.** Scatter plot demonstrates that self-transitions of the default-mode state during post-encoding rest (not pre-encoding rest) show the strongest correlation with memory performance, suggesting that stability of this consolidation-related network configuration is critical for memory outcomes. Dashed lines indicate 95% confidence intervals; solid lines show best linear fit. Error bars represent standard error. Notes: *$p < 0.05$, ***$p < 0.001$.
